# Absolute Positioning Accuracy Improvement in an Industrial Robot

**DOI:** 10.3390/s20164354

**Published:** 2020-08-05

**Authors:** Yizhou Jiang, Liandong Yu, Huakun Jia, Huining Zhao, Haojie Xia

**Affiliations:** School of Instrument Science and Opto-Electronics Engineering, Hefei University of Technology, Hefei 230009, China; jiangyizhou1@mail.hfut.edu.cn (Y.J.); huakun_jia@mail.hfut.edu.cn (H.J.); hnzhao@mail.hfut.edu.cn (H.Z.); hjxia@hfut.edu.cn (H.X.)

**Keywords:** absolute positioning accuracy, industrial robot, neural network, differential evolution algorithm

## Abstract

The absolute positioning accuracy of a robot is an important specification that determines its performance, but it is affected by several error sources. Typical calibration methods only consider kinematic errors and neglect complex non-kinematic errors, thus limiting the absolute positioning accuracy. To further improve the absolute positioning accuracy, we propose an artificial neural network optimized by the differential evolution algorithm. Specifically, the structure and parameters of the network are iteratively updated by differential evolution to improve both accuracy and efficiency. Then, the absolute positioning deviation caused by kinematic and non-kinematic errors is compensated using the trained network. To verify the performance of the proposed network, the simulations and experiments are conducted using a six-degree-of-freedom robot and a laser tracker. The robot average positioning accuracy improved from 0.8497 mm before calibration to 0.0490 mm. The results demonstrate the substantial improvement in the absolute positioning accuracy achieved by the proposed network on an industrial robot.

## 1. Introduction

Although industrial robots are flexible platforms and provide high repeatability for the automation of a variety of manufacturing tasks, a low absolute positioning accuracy may limit their applicability [1]. Error sources in robots can be either kinematic or non-kinematic [2,3,4]. Manufacturing and assembly tolerances cause deviation of the actual kinematic parameters from their nominal values, causing kinematic errors [5]. On the other hand, non-kinematic errors are usually neglected by their difficult modeling. Nevertheless, non-kinematic errors caused by factors such as temperature variations, joint and link compliance, and gear backlash have a considerable effect on the absolute positioning accuracy. Thus, an efficient method to effectively compensate positioning deviations caused by both kinematic and non-kinematic errors should be devised.

Extensive reports have shown that kinematic errors account for about 90% of the total positioning error [6]. Therefore, kinematic calibration effectively improves the absolute positioning accuracy of robots. This type of calibration comprises four steps: modeling, measurement, identification, and compensation or correction [7]. Thus, the kinematic model plays a critical role during robot calibration and should meet the following requirements for parameter identification: continuity, completeness, and minimality [8]. The Denavit–Hartenberg convention is a widely used modeling method to describe the robot kinematics [9]. However, this method is not continuous when two adjacent joint axes are parallel or nearly parallel, thus presenting singularities. To overcome the singularity problem, many studies have suggested alternative models. For instance, Hayati added a revolute parameter to the Denavit–Hartenberg convention, establishing the modified Denavit–Hartenberg model [10]. The complete and parametrically continuous model proposed by Meng and Zhuang provides completeness and continuity, but some of its parameters are redundant [11]. The product of exponentials (POE) model provides a complete geometric and parameterized representation of the robot motion, greatly simplifying the kinematic analysis of robotic mechanisms. The POE formula satisfies continuity, completeness, and minimality, being suitable and widely used for robot kinematic modeling [12].

Robot calibration aims to optimize an objective function and obtain accurate kinematic parameters [13]. The most common calibration algorithms are those based on the least squares method, such as the Gauss–Newton and Levenberg–Marquardt (LM) algorithms, with the latter being robust against interference and enabling global search [14,15]. However, neglecting error sources such as temperature variations and gear backlash limits the effectiveness and accuracy of robot calibration. Various correction methods have been developed to mitigate the influence of non-kinematic errors. Ma et al. demonstrated the importance of non-kinematic errors and generalized them by a representation with error matrices containing high-order Chebyshev polynomials that reflect individual error terms [16]. Whitney et al. developed a forward calibration method using joint encoder offset, link length, and consecutive-axis relative orientations as parameters and experimentally evaluated the effects of joint backlash, gear transmission, and compliance errors [17]. Chen et al. parameterized the joint flexibility error for estimation to improve the absolute positioning accuracy. However, the mathematical modeling of these methods is complex and various non-kinematic errors are ignored, limiting their applicability [18].

Given the difficulty to model non-kinematic errors, artificial neural networks have been used as an alternative to compensate the absolute positioning error [19]. Neural networks allow one to compensate positioning errors, avoid complex modeling, and comprehensively consider the influence of every error source. Ding et al. proposed a neural network combining with Faugeras vision system calibration technology for accurate calibration of a delta-robot vision system [20]. Likewise, Jang et al. used a radial basis function network to approximate the relationship between the robot joint readings and corresponding position errors [21]. Wang et al. developed a neural network to extract local features of error surface and estimated the positioning errors during calibration of a robot manipulator [22].

The performance of neural networks is influenced by their structure and parameters. To optimize neural networks, Rouhani et al. introduced one-pass heuristic rules to determine the center, number, and spread of hidden neurons in the network [23]. Feng et al. used evolutionary particle swarm optimization and developed a neural network with a self-generating radial basis function [24]. González et al. developed a multiobjective evolutionary algorithm to optimize neural networks. Differential evolution algorithms enable global optimization with high identification accuracy and optimal rate [25]. Accordingly, we adopt differential evolution for pre-training of a neural network to optimize the thresholds, initial weights, and the number of hidden neurons for maximizing accuracy and efficiency. We conducted simulations and experiments to verify the performance of the proposed network by employing a six-degree-of-freedom (DOF) industrial robot and a laser tracker as reference.

The main contributions of this work can be summarized as follows. (1) The influence of kinematic and non-kinematic errors on the absolute positioning accuracy is analyzed, and the limitations of kinematic calibration are addressed. (2) A neural network optimized using differential evolution is proposed to enhance the absolute positioning accuracy. Thresholds, initial weights, and the number of hidden neurons in the neural network are optimized using differential evolution to improve the performance of the neural network. The proposed network mitigates the effects of kinematic and non-kinematic errors and thus improves the absolute positioning accuracy of a robot. (3) The experiment and simulation are completed with the six-DOF robot as the research object and the laser tracker as the measuring instrument. The theoretical correctness and effectiveness of the proposed neural network are verified by simulations and experiments.

The remainder of this paper is organized as follows: The influences of kinematic and non-kinematic errors on robot positioning accuracy are analyzed in Section 2. The proposed neural network optimized by differential evolution is detailed in Section 3. In Section 4, simulations and experiments are performed for compensating the absolute positioning error. The discussion and conclusions are presented in Section 5.

## 2. Kinematic and Non-Kinematic Error Analysis

**Figure 1:**
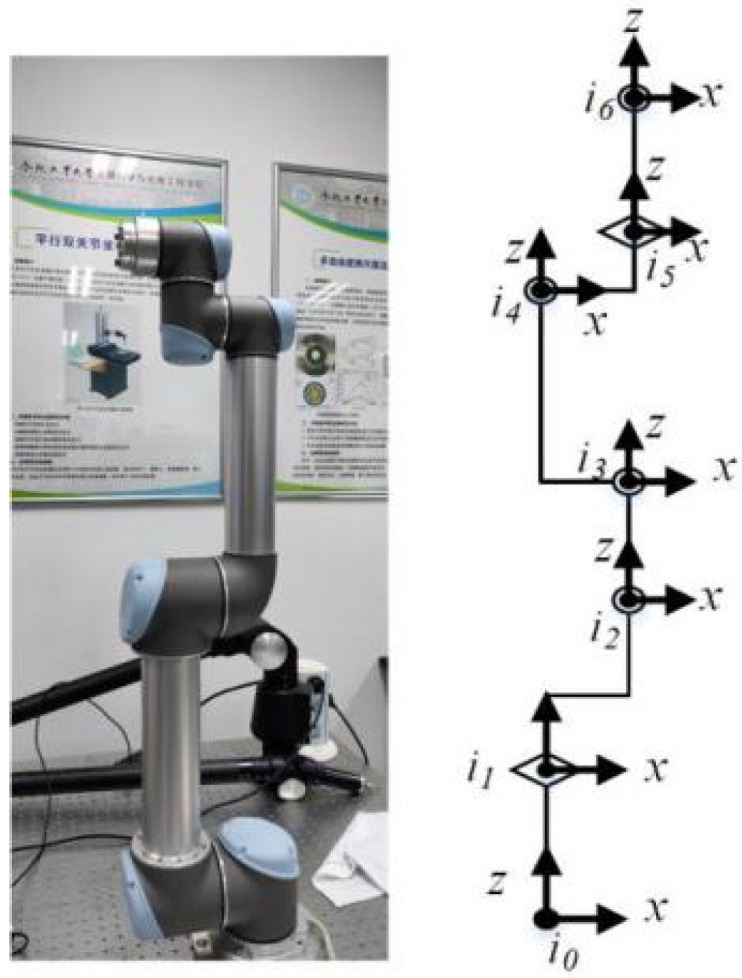
Coordinate systems of the robot manipulator.

The accuracy of a robot kinematic model depends on robot calibration. We use the POE model to describe the robot kinematics [26], considering the coordinate systems of the robot shown in Figure 1. 

The pose of end-effector frame {t} with respect to base frame {s} is given by

(1) $$g_{st}(\theta )=e^{\hat{\xi_{1}}\theta_{1}}e^{\hat{\xi_{2}}\theta_{2}}e^{\hat{\xi_{3}}\theta_{3}}e^{\hat{\xi_{4}}\theta_{4}}e^{\hat{\xi_{5}}\theta_{5}}e^{\hat{\xi_{6}}\theta_{6}}e^{\hat{\xi_{st}}}=\left[\begin{array}{cc} p(\theta ) & r(\theta )\\ 1 & 1 \end{array}\right]=\left[\begin{array}{cccc} n_{x} & o_{x} & a_{x} & r_{x}\\ n_{y} & o_{y} & a_{y} & r_{y}\\ n_{z} & o_{z} & a_{z} & r_{z}\\ 0 & 0 & 0 & 1 \end{array}\right]$$

where $\theta_{i}$ indicates the robot joint variable and ${\hat{\xi }}_{st}$ is the twist of the initial transformation. In Equation (1), ${\hat{\xi }}_{i}$ can be expressed as

(2) $${\hat{\xi }}_{i}=\left[\begin{array}{cc} {\hat{w}}_{i} & v_{i}\\ 0 & 0 \end{array}\right]$$

where ${\hat{w}}_{i}$ and $v_{i}$ are given by

(3) $${\hat{w}}_{i}=\left[\begin{array}{ccc} 0 & -w_{zi} & w_{yi}\\ w_{zi} & 0 & -w_{xi}\\ -w_{yi} & w_{xi} & 0 \end{array}\right]$$

(4) $$v_{i}=-w_{i}\times q_{i}$$

where $q_{i}=[q_{xi},q_{yi},q_{zi}]$ represents the coordinate of the origin of each axis in the base coordinate system {s}; $w_{i}=[w_{xi},w_{yi},w_{zi}]$ is the direction vector in system {s} of each rotation axis.

For a rotational joint, the exponential matrix of the motion screw can be represented as

(5) $$e^{\hat{\xi_{i}}\theta_{i}}=\left[\begin{array}{cc} e^{{\hat{w}}_{i}\theta_{i}} & \left(I-e^{{\hat{w}}_{i}\theta_{i}})(w_{i}\times (-w_{i}\times q_{i})\right)\\ 0 & 1 \end{array}\right]$$

The forward kinematics of a six-DOF serial robot is given by

(6) $$f_{c}=g(w_{1},\cdots ,w_{6},q_{1},\cdots ,q_{6},\theta )=e^{{\hat{\xi }}_{1}\theta_{1}}e^{{\hat{\xi }}_{2}\theta_{2}}\cdots e^{{\hat{\xi }}_{6}\theta_{6}}e^{{\hat{\xi }}_{st}}$$

**Figure 2:**
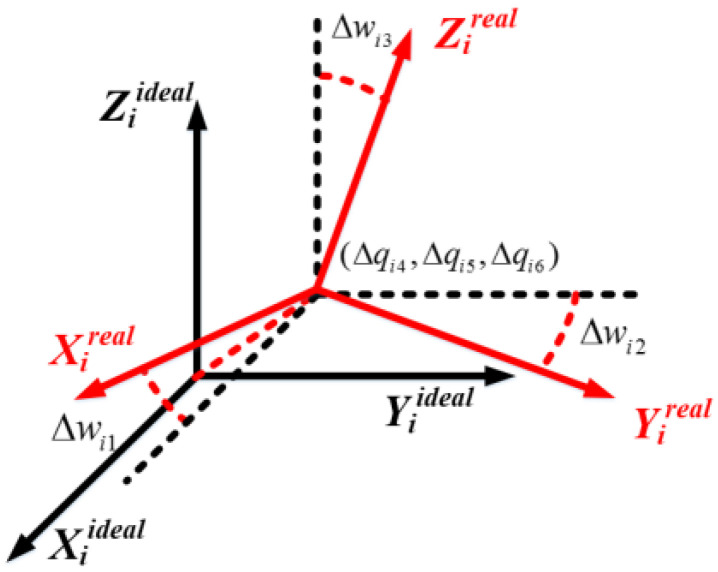
Definition of kinematic errors for link *i*.

The kinematic parameters of the robot generally deviate from their designed values due to different kinematic errors such as mechanical deformation, assembly and machining errors etc., as shown in Figure 2.

When the kinematic errors are only considered, the actual robot position $f_{real}$ can be expressed as

(7) $$f_{real}=g(w_{1}+\Delta w_{1},\cdots ,w_{6}+\Delta w_{6},q_{1}+\Delta q_{1},\cdots ,q_{6}+\Delta q_{6},\theta )$$

where $\Delta q_{i}$ and $\Delta w_{i}$ represent the kinematic errors to be corrected. The mathematical expression of kinematic errors can be obtained by kinematic modeling. These errors exist whether the robot is moving or not. Therefore, kinematic errors can be identified by the calibration method so as to effectively improve the positioning accuracy of the robot. 

However, non-kinematic errors undermine accuracy, but they are generally neglected due to their modeling difficulty and complexity. All error sources whose contributions to positioning errors cannot be characterized by kinematic parameters are herein described as non-kinematic errors [18]. The non-kinematic errors relate to pose and dynamical behavior of the robot, so it is difficult to obtain the exactly mathematical expression. Therefore, calibration cannot mitigate the effects of non-kinematic errors. Non-kinematic errors with considerable effects, such as those related to strain wave gearing and joint flexibility, occur between the angular encoder and output shaft at the joint, and the nominal joint variable should be compensated for computing the actual joint rotation. 

Considering non-kinematic errors, the actual robot joint variable can be expressed as

(8) $$\theta_{i}{}_{\_real}={\hat{\theta }}_{i}+C_{i}({\hat{\theta }}_{i})$$

where ${\hat{\theta }}_{i}$ is the nominal robot joint variable and $C_{i}({\hat{\theta }}_{i})$ represents the compensation factor for non-kinematic errors.

We can use Chebyshev polynomials to calculate $C_{i}({\hat{\theta }}_{i})$ [16]:

(9) $$C_{i}({\hat{\theta }}_{i})=a_{0}+a_{1}c_{1}({\hat{\theta }}_{i})+\cdots +a_{m}c_{m}({\hat{\theta }}_{i})$$

with

(10) $$\begin{array}{l} c_{0}({\hat{\theta }}_{i})=1,\;c_{1}({\hat{\theta }}_{i})=\lambda ,\;c_{2}({\hat{\theta }}_{i})=2{\hat{\theta }}_{i}{}^{2}-1,\;c_{3}({\hat{\theta }}_{i})=4{\hat{\theta }}_{i}{}^{3}-3{\hat{\theta }}_{i},\\ c_{4}({\hat{\theta }}_{i})=8{\hat{\theta }}_{i}{}^{4}-8{\hat{\theta }}_{i}{}^{2}+1\;,\cdots ,\;c_{m+1}({\hat{\theta }}_{i})=2{\hat{\theta }}_{i}c_{m}({\hat{\theta }}_{i})-c_{m-1}({\hat{\theta }}_{i}) \end{array}$$

where *m* denotes the order of the Chebyshev polynomial and $a_{0},a_{1},\cdots ,a_{m}$ are the polynomial coefficients to be calculated. 

Thus, when nominal joint variables and theoretical kinematic parameters of a robot are known, its actual position is given by

(11) $$f_{real}=g(\Delta w_{1},\cdots ,\Delta w_{6},\Delta q_{1},\cdots ,\Delta q_{6},C_{1}({\hat{\theta }}_{1}),\cdots ,C_{6}({\hat{\theta }}_{6}))$$

The complexity of non-kinematic errors and the coupling between the compensation of nominal joint variables and robot pose hinder the determination of the actual mathematical representation of the robot position. Therefore, we use a neural network to directly estimate the robot position based on the joint variables. This method avoids complex calibration and modeling, effectively improves the absolute positioning accuracy of the robot and mitigates the influence of kinematic and non-kinematic errors.

## 3. The Proposed Neural Network

Different neural networks such as radial basis function and back-propagation networks have been widely used to compensate the absolute positioning error [19,20,21,22]. The compensation results depend on the network structure and parameters including thresholds, initial weights, and number of hidden neurons. Thus, we adopt differential evolution to optimize the structure and parameters of the proposed neural network for maximizing the compensation effect.

### 3.1. The Back-Propagation Neural Network

The back-propagation neural network is a kind of artificial neural network trained by the error back-propagation method. Its outstanding advantages are its strong linear mapping ability and flexible network structures. They provide strong linear mapping and a flexible structure. 

**Figure 3:**
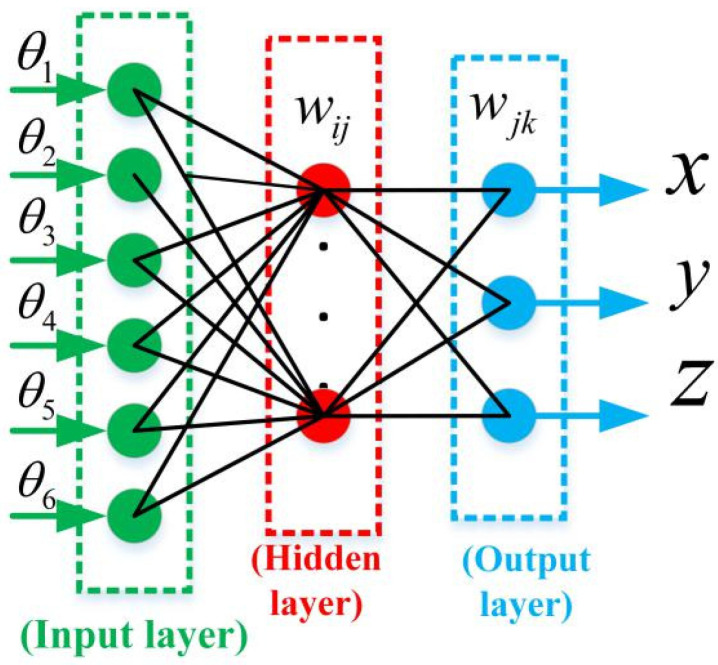
Structure of the back-propagation neural network.

Figure 3 shows the proposed neural network that consists of input, hidden, and output layers. In the proposed network, the input layer has six nodes representing the robot joint variables, and the output layer has three nodes representing the coordinates of the robot position obtained from the laser tracker.

In Figure 3, $w_{ij}$ is the connection weight between neuron *i* in the input layer and neuron *j* in the hidden layer, and $w_{jk}$ is that from neuron *j* in the hidden layer to neuron *k* in the output layer. The output from the hidden layer is expressed as

(12) $$H_{j}=G(\sum_{i=1}^{6}w_{ij}\theta_{i})\;j=1,2,\cdots ,l$$

where *l* is the number of nodes in the hidden layer and *G* is an activation function, which we set as follows:

(13) $$G(x)=\frac{1}{1+e^{x}}$$

For *x*, the output layer provides

(14) $$x=\sum_{j=1}^{l}H_{j}w_{jx}$$

The residual errors are obtained by subtracting the predicted values from the expected ones, which can be considered as position error values. The residual mean squared error is given by

(15) $$F=\frac{1}{m}\sum_{v=1}^{m}{\left(T_{v}-O_{v}\right)}^{2}\begin{array}{cc} & v=1,2,\cdots ,m \end{array}$$

where *m* is the number of training samples, $O_{v}=[x_{v},y_{v},z_{v}]$ is the neural network output, and $T_{v}$ is the expected output.

As long as the residual mean squared error is equal to or higher than the target training error or the number of iterations does not reach its limit, the biases and weights are updated to reduce the deviation between the predicted and expected values. Using gradient descent, the weights are updated as follows:

(16) $$\Delta w_{ij}=-\eta \frac{\partial F}{\partial w_{ij}}$$

(17) $$w_{ij}=\Delta w_{ij}+w_{ij}$$

**Figure 4:**
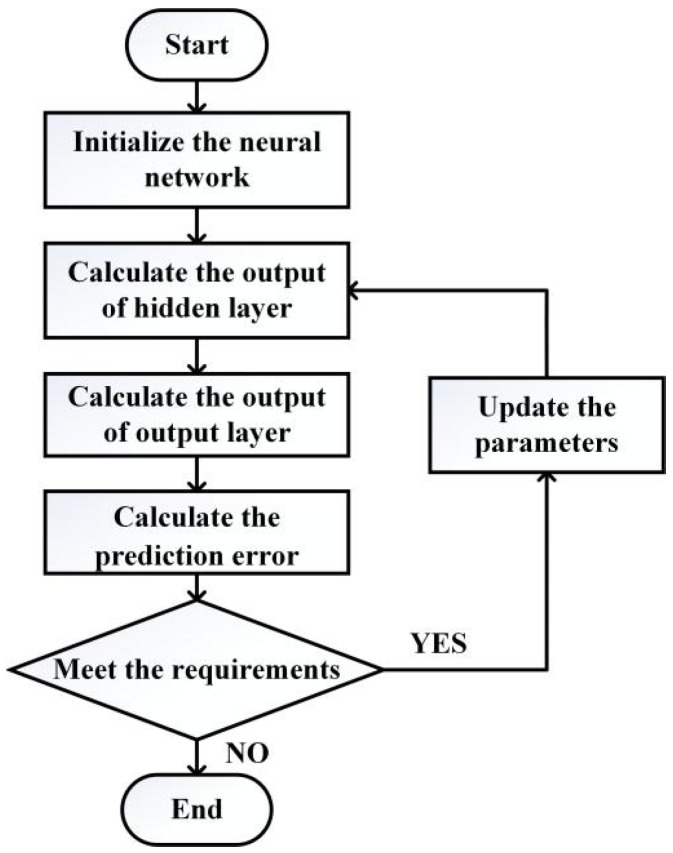
Training flowchart of the neural network.

Figure 4 shows the training flowchart of the neural network.

### 3.2. Differential Evolution Optimization

Differential evolution allows one to perform global optimization with high identification accuracy and optimal rate. Differential evolution is a population-based stochastic optimizer that explores the search space by sampling from multiple random initial points [27]. We use differential evolution to pre-train the proposed neural network for optimizing its thresholds, initial weights, and number of hidden neurons. 

The differential evolution algorithm typically consists of three elements:

(1) Encoding solution population;
(2) Fitness function to evaluate solution optimality;
(3) Evolutionary operation comprising selection, crossover and mutation.

Individual $\varepsilon_{i}=\{w_{i},B_{i}\}$ is initialized to represent different numbers of hidden layers, weight values $w_{i}$, and threshold values $B_{i}$. The length of $\varepsilon_{i}$ is obtained from the number of hidden neurons and calculated as follows:

(18) $$length(\varepsilon_{i})=n_{in}n_{hid}+n_{hid}+n_{hid}n_{out}+n_{out}$$

where $n_{in}$, $n_{hid}$ and $n_{out}$ are the number of neurons in the hidden, input, and output layers, respectively. Different values of $\varepsilon_{i}$ determine the performance of the neural network, as few neurons undermine accuracy, whereas many neurons increase the training time and lead to overfitting. In addition, the initial weights strongly affect the performance and convergence rate of the neural network. Hence, differential evolution allows one to enhance the learning rate and prediction accuracy.

For differential evolution, mutation is expressed as

(19) $$\varepsilon_{mi}^{g}=\varepsilon_{gbest}^{g}+0.5(\varepsilon_{a}^{g}-\varepsilon_{b}^{g}).$$

where *g* is the number of iterations, $\varepsilon_{gbest}^{g}$ is the neural network parameter providing the highest performance, and $\varepsilon_{a}^{g}$ and $\varepsilon_{b}^{g}$ are two particles randomly selected from the population for differential evolution. As the lengths of $\varepsilon_{gbest}^{g}$, $\varepsilon_{a}^{g}$, and $\varepsilon_{b}^{g}$ are different due to the varying number of hidden neurons, mutation cannot be directly performed. Instead, we apply the best selection method to guarantee the same vector lengths during mutation. Specifically, the length of the best individual, $\varepsilon_{gbest}^{g}$, is employed as the basis to unify the lengths of the other individuals and perform mutation. When $length(\varepsilon_{a}^{g})<length(\varepsilon_{gbest}^{g})$, the missing parameters are randomly added to $\varepsilon_{a}^{g}$ to obtain $length(\varepsilon_{a}^{g})=length(\varepsilon_{gbest}^{g})$. When $length(\varepsilon_{a}^{g})>length(\varepsilon_{gbest}^{g})$, the excess parameters of $\varepsilon_{a}^{g}$ are considered as interferent and disregarded during mutation.

Test individuals $\varepsilon_{T}$ are then generated through crossover using Equation (20), CR is a real-valued crossover probability factor in range [0,1] that controls the probability that a trial vector parameter will be randomly chosen. Generally, CR affects the convergence velocity and robustness of the search process. In this paper, CR = 0.9 to ensure a fast convergence rate.

Thus, for each parameter of the particle, crossover can be expressed as

(20) $$\varepsilon_{T}(j)=\left\{ \begin{array}{l} \varepsilon_{mi}^{g}(j),rand(0,1)\leq CR\\ \varepsilon_{i}^{g}(j),rand(0,1)>CR \end{array}\right.\left(j=1,2,\cdots ,n_{hid}\right)$$

Selection is based on a greedy search strategy expressed as

(21) $$\varepsilon_{i}^{g+1}=\{\begin{array}{l} \varepsilon_{T},F(\varepsilon_{T})<F(\varepsilon_{i}^{g})\\ \varepsilon_{i}^{g},F(\varepsilon_{T})>F(\varepsilon_{i}^{g}) \end{array}$$

**Figure 5:**
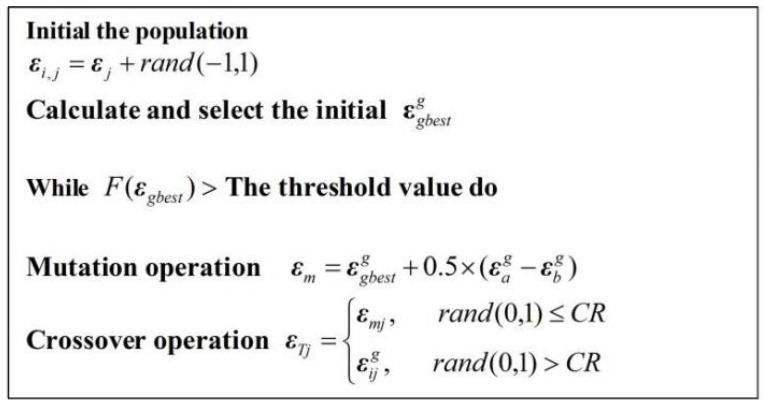
Differential evolution for pre-training the proposed neural network.

The optimized parameters during pre-training are applied to the proposed neural network. If the predicted values after pre-training do not reach the expected deviation, the number of hidden layers of each particle is updated using Equation (22). This procedure is repeated until the desired values are obtained.

(22) $$K_{i}=\{\begin{array}{l} K_{i}-1\begin{array}{cc} & if(K_{best}<K_{i}) \end{array}\\ K_{i}+1\begin{array}{cc} & if(K_{best}\geq K_{i}) \end{array} \end{array}$$

where $K_{i}$ is the number of hidden layers for individual *i* and $K_{best}$ is the best solution. The differential evolution algorithm is described in Figure 5 and proceeds until the desired prediction error is reached.

## 4. Simulations and Experiments

We validated the performance of the proposed neural network through simulations and experiments of error compensation on a six-DOF robot manipulator. For comparison, the robot kinematic parameters were calibrated using the POE model [12] and LM algorithm [14,15].

**Figure 6:**
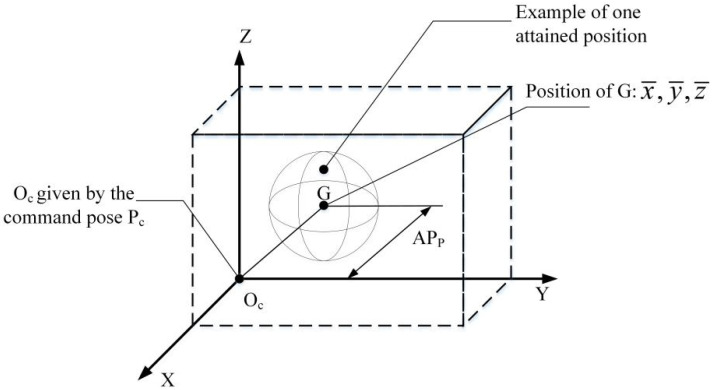
Positioning accuracy.

As described in standard ISO 9283 [28], the positioning accuracy is the difference between the position of a command pose and the barycenter of the attained positions, as illustrated in Figure 6.

Thus, the positioning accuracy can be calculated as

(23) $$AP_{P}=\sqrt{{\left(\bar{x}-x_{c}\right)}^{2}+{\left(\bar{y}-y_{c}\right)}^{2}+{\left(\bar{z}-z_{c}\right)}^{2}}$$

with

(24) $$\bar{x}=\frac{1}{n}\sum_{j=1}^{n}x_{j},\bar{y}=\frac{1}{n}\sum_{j=1}^{n}y_{j},\bar{z}=\frac{1}{n}\sum_{j=1}^{n}z_{j}$$

where $\bar{x},\bar{y},\bar{z}$ are the coordinates of the barycenter of the cluster of points obtained after executing the same pose *n* times, $x_{c},y_{c},z_{c}$ are the coordinates of the command pose, and $x_{j},y_{j},z_{j}$ are the coordinates of the *j*-th attained pose along the respective axes.

**Figure 7:**
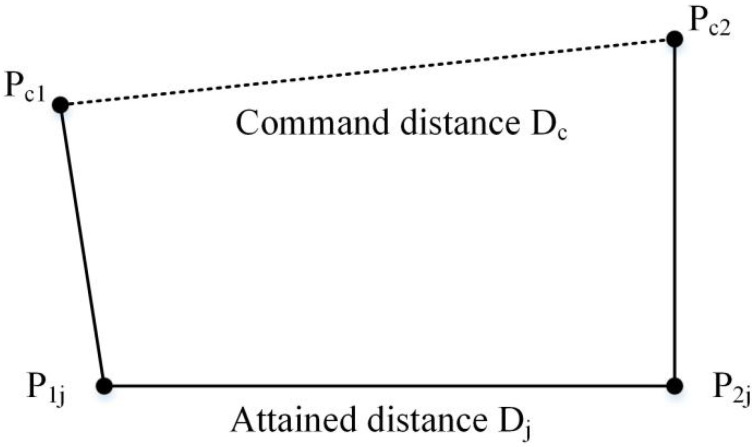
Distance accuracy.

Analogously, the distance accuracy expresses the deviation in positioning and orientation between the command distance and mean of the attained distances, as illustrated in Figure 7.

The distance accuracy can be calculated as

(25) $$AP_{d}=\bar{D}-D_{c}$$

with

(26) $$\bar{D}=\frac{1}{n}\sum_{j=1}^{n}D_{j}$$

(27) $$D_{j}=\left|P_{1j}-P_{2j}\right|=\sqrt{{\left(x_{1j}-x_{2j}\right)}^{2}+{\left(y_{1j}-y_{2j}\right)}^{2}+{\left(z_{1j}-z_{2j}\right)}^{2}}$$

(28) $$D_{c}=\left|P_{c1}-P_{c2}\right|=\sqrt{{\left(x_{c1}-x_{c2}\right)}^{2}+{\left(y_{c1}-y_{c2}\right)}^{2}+{\left(z_{c1}-z_{c2}\right)}^{2}}$$

We use both the distance accuracy and positioning accuracy to quantify error compensation.

### 4.1. Simulations

To perform simulations, we added random parameter errors to the theoretical robot kinematic parameters as actual parameters and non-kinematic errors as the compensation of the nominal joint variables of the robot. The actual and theoretical end-effector position coordinates of the robot were calculated using the POE model.

**Figure 8:**
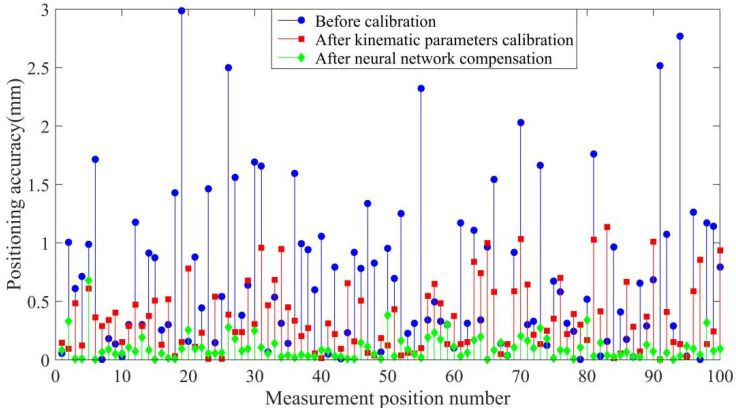
Positioning accuracy of the simulations.

**Figure 9:**
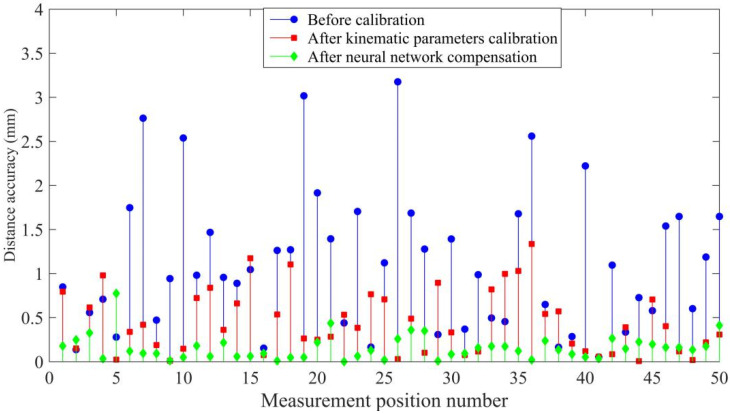
Distance accuracy of the simulations.

We applied 1000 pairs of joint variables and position coordinates to optimize the thresholds, initial weights, and number of hidden neurons in the proposed network using differential evolution. The same 1000 samples were applied to train the optimized neural network, and 100 command points were employed for verification of the prediction accuracy of the trained neural network. In addition, the robot kinematic parameters were calibrated using the POE model and LM algorithm for comparison by applying the same samples. The compensation results are shown in Figure 8 and Figure 9.

Compared to the calibration method, the proposed neural network optimized using differential evolution provides better compensation. The maximum distance accuracy is improved from 3.1760 mm to 0.7743 mm, and the average distance accuracy is improved from 1.1118 mm to 0.1564 mm by using the proposed network. The maximum positioning accuracy is improved from 3.1760 mm to 0.7743 mm and the average positioning accuracy is improved from 0.7411 mm to 0.1007 mm by using the proposed algorithm.

As summarized in Table 1 and Table 2, the proposed method has the best absolute positioning accuracy and distance accuracy.

### 4.2. Experiment

**Figure 10:**
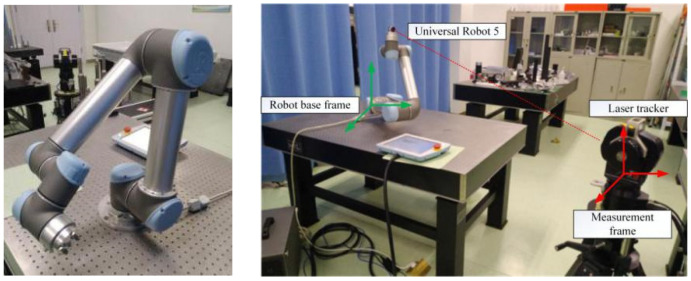
The experimental setup.

The calibration system, as shown in Figure 10, consists of a six-DOF serial robot (Universal Robot 5, Universal Robots), a Laser Tracker (API T3, Automated Precision Inc., Maryland, USA) with an accuracy of 0.005 mm/m, and an accompanying laser reflector. The reflector was fixed at an assigned location on the robot end-effector. The robot moved within the workspace and spatial position coordinates of the end-effector data were collected using an laser tracker as the output of the neural network. Additionally, the corresponding joint angles of the sampling points were recorded as the input of the neural network.

**Figure 11:**
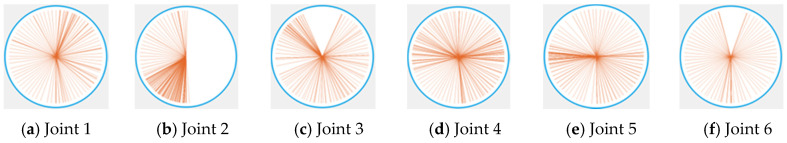
Angular distribution covering the joint space.

We measured 600 samples in the workspace of the robot for optimization and training of the proposed network. The same samples were used for calibration of kinematic parameters using the POE model and LM algorithm. The samples were selected such that the corresponding joint variables covered the robot joint working ranges, as shown in Figure 11. In addition, 100 command points in the robot workspace were randomly selected to verify the accuracy of the calibration methods and proposed network.

**Figure 12:**
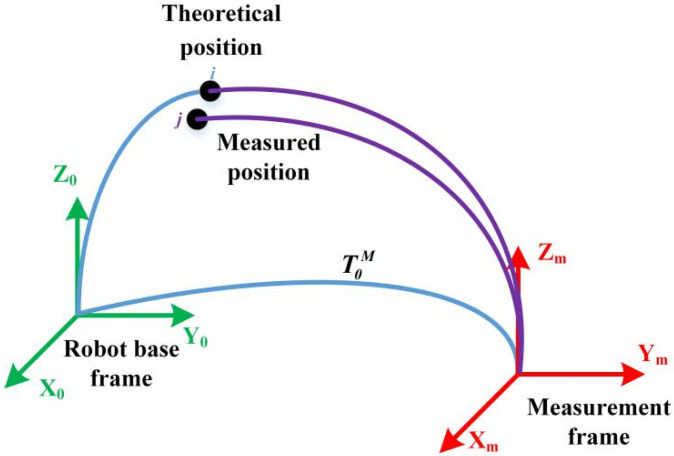
Experiment coordinate systems considering the measurement and robot base.

Figure 12 shows the robot base and measurement coordinate systems considered in the experiment. Gan et al. presented a simple but effective calibration of the relative rotation matrix and translation vector for converting the base frames of two coordinate systems using quaternions [29]. We used this method to determine the transformation between the measurement and robot base frames. The standard position coordinates measured using the laser tracker can be expressed into the robot base coordinate system. Therefore, we conducted the prediction and training of the proposed network by integrating robot joint variables with exact positioning with respect to the robot base.

**Figure 13:**
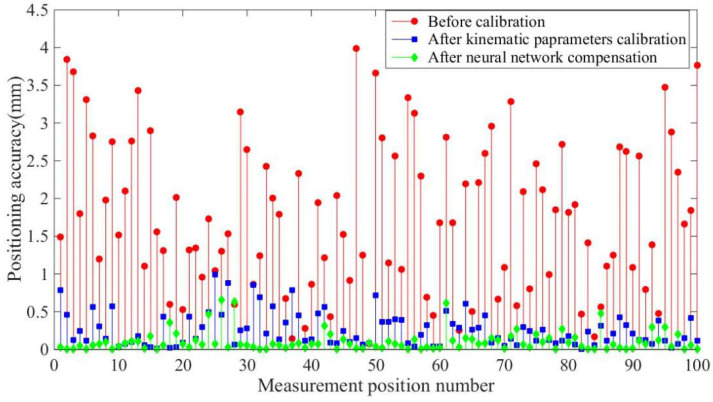
Positioning accuracy of the experiments.

**Figure 14:**
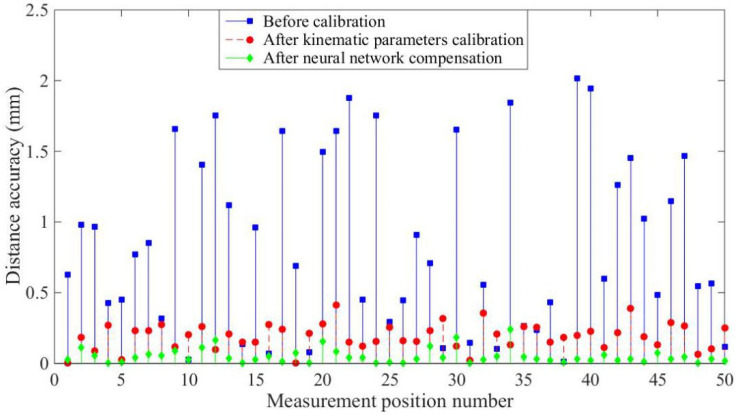
Distance accuracy of the experiments.

Figure 13 and Figure 14 and Table 3 and Table 4 show the experimental results, with the optimized network accurately predicting the position coordinates. Before calibration, the maximum positioning accuracy is 3.1760 mm and the maximum distance accuracy 1.1118 mm, which is affected by kinematic and non-kinematic errors. After calibration, using the POE model and the LM algorithm, the positioning accuracy and distance accuracy of the robot were significantly improved, which is due to the reduction in kinematic errors through calibration. However, neglecting error sources such as temperature variations and gear backlash limits the effectiveness and accuracy of robot calibration. Therefore the proposed network provides the best absolute positioning accuracy and distance accuracy because the proposed network mitigates the effects of kinematic and non-kinematic errors. After error compensation, the maximum positioning accuracy is improved from 3.1760 mm to 0.7743 mm and the average positioning accuracy is improved from 1.1118 mm to 0.1564 mm by using the proposed algorithm. The maximum distance accuracy is improved from 2.0140 mm to 0.2392 mm and the average distance accuracy is improved from 0.8497 mm to 0.0493 mm.

## 5. Discussion and Conclusions

The absolute positioning accuracy of robots has become increasingly important to support their widespread applications, particularly when offline programming is required. Error sources in robots can be classified into kinematic and non-kinematic errors. Conventional calibration can reduce the influence of kinematic errors on positioning accuracy, but the complexity of non-kinematic error sources and modeling hinders the compensation of such errors.

We applied a neural network to compensate both kinematic and non-kinematic errors with the aim of improving the absolute positioning accuracy of robot manipulators. The proposed network can avoid complex modeling while comprehensively considering the influence of all error sources. As the performance of a neural network is influenced by its structure and parameters, we optimized the proposed network using differential evolution. The thresholds, initial weights, and number of hidden neurons in the network are optimized using differential evolution to improve efficiency and performance. Using the proposed network, the effects of kinematic, and non-kinematic errors decreased, thus improving the absolute positioning accuracy of an industrial robot.

The theoretical correctness and effectiveness of the proposed method are verified by simulations and experiments on a six-DOF serial robot and compared with calibration using the POE model and LM algorithm. The absolute positioning accuracy improves using the proposed network, and the experimental results verify the correctness and effectiveness of the network. After calibration, the robot average distance accuracy improved from 0.8497 mm before calibration to 0.04933 mm. Likewise, the robot average positioning accuracy improved from 3.176 mm before calibration to 0.7743 mm.

## Figures and Tables

**Table 1:** Positioning accuracy of the simulations.

Positioning Accuracy (mm)	Mean Error	Max Error
Before calibration	0.7411	2.9500
After kinematic parameter calibration	0.3611	1.1379
After neural network compensation	0.1007	0.6803

**Table 2:** Distance accuracy of the simulations.

Distance Accuracy (mm)	Mean Error	Max Error
Before calibration	1.1180	3.1760
After kinematic parameter calibration	0.4461	1.3360
After neural network compensation	0.1564	0.7743

**Table 3:** Positioning accuracy of the experiments.

Positioning Accuracy (mm)	Mean Error	Max Error
Before calibration	1.7730	3.9930
After kinematic parameter calibration	0.2595	0.9877
After neural network compensation	0.1041	0.6559

**Table 4:** Distance accuracy of the experiments.

Distance Accuracy (mm)	Mean Error	Max Error
Before calibration	0.8497	2.0140
After kinematic parameter calibration	0.1915	0.4113
After neural network compensation	0.0493	0.2392
